# Lysine Acetylation is an Important Post-Translational Modification that Modulates Heat Shock Response in the Sea Cucumber *Apostichopus japonicus*

**DOI:** 10.3390/ijms20184423

**Published:** 2019-09-09

**Authors:** Dongxue Xu, Xuan Wang

**Affiliations:** 1 College of Marine Science and Engineering, Qingdao Agricultural University, Qingdao 266109, China; 2 Key Laboratory of Mariculture (Ministry of Education), Ocean University of China, Qingdao 266003, China

**Keywords:** *Apostichopus japonicus*, heat stress, lysine acetylation, lysine acetyltransferases (KATs), chaperones, ribosome

## Abstract

Heat stress (HS) is an important factor for the survival of the marine organism *Apostichopus japonicus*. Lysine acetylation is a pivotal post-translational modification that modulates diverse physiological processes including heat shock response (HSR). In this study, 4028 lysine acetylation sites in 1439 proteins were identified in *A. japonicus* by acetylproteome sequencing. A total of 13 motifs were characterized around the acetylated lysine sites. Gene Ontology analysis showed that major acetylated protein groups were involved in “oxidation–reduction process”, “ribosome”, and “protein binding” terms. Compared to the control group, the acetylation quantitation of 25 and 41 lysine sites changed after 6 and 48 h HS. Notably, lysine acetyltransferase CREB-binding protein (CBP) was identified to have differential acetylation quantitation at multiple lysine sites under HS. Various chaperones, such as caseinolytic peptidase B protein homolog (CLBP), T-complex protein 1 (TCP1), and cyclophilin A (CYP1), showed differential acetylation quantitation after 48 h HS. Additionally, many translation-associated proteins, such as ribosomal proteins, translation initiation factor (IF), and elongation factors (EFs), had differential acetylation quantitation under HS. These proteins represented specific interaction networks. Collectively, our results offer novel insight into the complex HSR in *A. japonicus* and provide a resource for further mechanistic studies examining the regulation of protein function by lysine acetylation.

## 1. Introduction

Post-translation modifications (PTMs) are as important as the transcriptional ways in regulating protein activity [1]. PTMs can regulate allosteric effects of proteins and can affect binding sites and domains for protein–protein interactions [2]. PTMs consist of a variety of protein modifications, including phosphorylation, acetylation, ubiquitylation, methylation, and hydroxylation. Lysine acetylation, as a critical type of PTM, was first discovered in histone proteins [3]. Histone proteins interact with DNA and are the fundamental building blocks of eukaryotic chromatin. Lysine acetylation may direct the assembly of histones and help regulate the transcription of genes [4,5]. Outside of histones, there has been a rapid proliferation in the attention of nonhistone targets of lysine acetylation. For example, lysine acetylation is already extensively investigated in a wide range of transcription factors, molecular chaperones, cytoskeletal proteins, and nuclear import factors [6,7]. The acetylation of nonhistone proteins can regulate enzymatic activity, protein interactions, protein stability, and metabolic pathways [6,8].

Living organisms are frequently faced with abiotic stress conditions such as extreme temperatures, low nutrient availability, salinity, drought, and high ultraviolet irradiation. The physiology, behavior, and development of organisms are usually adjusted to assure the survival under changing conditions [9]. PTM allows a more rapid response than transcriptional regulation, thereby contributing to the ability of organisms to respond to stress rapidly [10]. Lysine acetylation, widespread in prokaryotes and eukaryotes, plays a crucial role in facilitating to mitigate the potential damage of environmental stresses [1]. In *Mycobacterium* spp., the universal stress protein (USP), which provides resistance to various stressors, can be acetylated at a single lysine residue [11]. Deletion of the sirtuin deacetylase in *Mycobacterium tuberculosis* results in increased resistance to heat stress [12]. In *Escherichia coli*, cells with decreased protein acetylation levels fail to resist against heat and oxidative stress [13]. That study also performed a whole-transcriptome analysis, which suggested many stress-related systems were repressed in the mutant, including genes related to heat shock, osmotic stress, acid resistance, cold shock, and carbon starvation [13]. In eukaryotes, lysine acetylation has also been shown to play crucial roles in signaling pathways of diverse stress responses, such as heat stress [14] and oxidative stress [15]. These studies further support the hypothesis that lysine acetylation is a mechanism for protection against environmental stresses.

The sea cucumber *Apostichopus japonicus* belongs to phylum Echinodermata and class Holothuroidea. Echinoderms are of special interest for studies because they can provide insights on the evolutionary origins of physiological responses and the organism–environment interface that occur in vertebrates [16]. The sea cucumber also plays an important ecological role in the ocean ecosystem by breaking down detritus and organic matter and recycling nutrients back into the water. *A. japonicus* is mainly distributed along the coast zones of northern China, southeastern Russia, Japan, Republic of Korea, and Democratic People’s Republic of Korea. In China, *A. japonicus* is a popular seafood, and the aquaculture industry flourishes. However, warming trends have led to serious consequences in the marine organisms [17]. A high mortality of *A. japonicus* occurs frequently in the coastal ocean and ponds in the summer because of the high temperature [18]. It is estimated that about 80% mortality occurred in the coastal ocean and ponds in the main distribution regions of *A. japonicus* in northern China in the summers of 2013 and 2016. In view of the evolutionary, ecological, and economic values of *A. japonicus*, understanding the heat shock response (HSR) of *A. japonicus* is of vital importance.

In this study, we aim to explore the acetylation profiling of proteins in *A. japonicus* and how lysine acetylation affects HSR in the species. A temperature of 26 °C is the typical temperature stress that sea cucumbers experience in summer in most coasts of northern China. Besides, our previous studies implied HSR of *A. japonicus* was activated at this temperature [19,20]. Many stress genes had a peak expression value after 6 h at 26 °C. On the other hand, some genes involved in metabolism started to significantly change after 48 h at 26 °C [21]. The two groups represented the starting and processing periods of HSR respectively. The intestine was sensitive to HS, which showed obvious changes of gene expression [19,20]. Therefore, we set our treatment groups as 6 and 48 h after 26 °C HS, and we sampled the intestine tissue in the present study.

## 2. Results

### 2.1. A. japonicus Had a Large Number of Acetylated Proteins and Sites by Proteome-Wide Analysis

**Figure 1:**
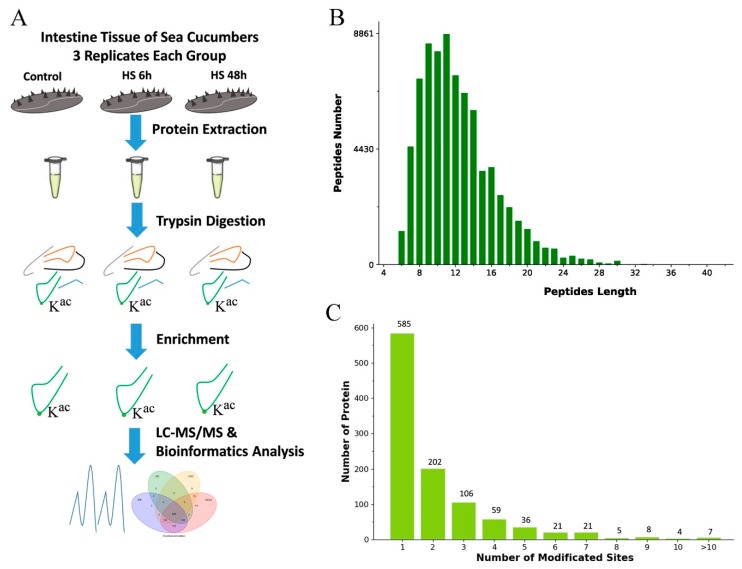
Proteome-wide identification of lysine acetylation sites in sea cucumbers. (**A**) Experimental strategy for quantifying lysine acetylation. (**B**) Peptide number and peptide length. (**C**) Number of proteins and number of modified sites.

To map lysine acetylation sites in *A. japonicus*, proteins were extracted and digested into peptides with trypsin as described in Materials and Methods (Figure 1A). The length of most acetylation peptides obtained was between 6 and 30 amino acids, and most peptides consisted of 8–16 amino acids (Figure 1B). Altogether, 4028 lysine acetylation sites in 1439 proteins were identified. We also analyzed the distribution of these acetylated sites. We identified 585 proteins that had one acetylated site, 202 proteins having two acetylated sites, and 106 proteins possessing three sites (Figure 1C).

### 2.2. 13 Acetylation Motifs Were Characterized in A. japonicus

**Figure 2:**
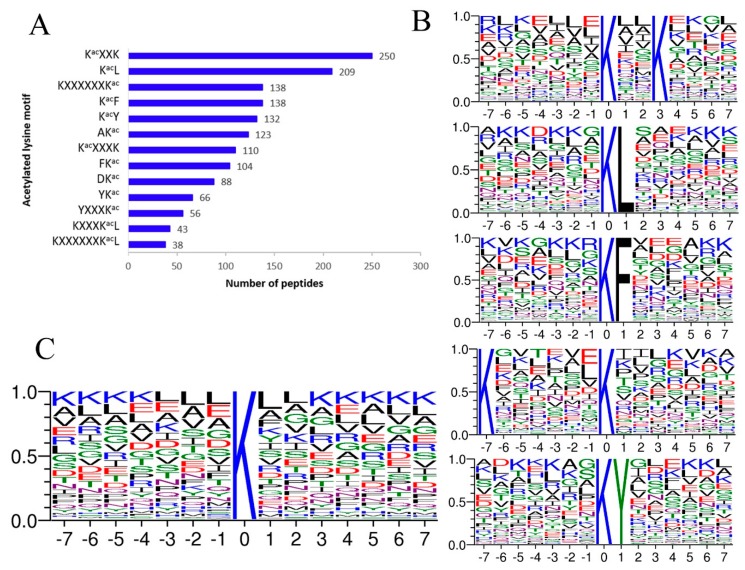
Properties of lysine acetylation sites. (**A**) A total of 13 enriched motifs and the number of according peptides. (**B**) Sequences of the top five motifs with the biggest probability. (**C**) Sequence probability logos of significantly enriched acetylation site motifs for ±7 amino acids around the lysine acetylation sites.

We identified acetylation motifs using motif-X software (Boston, MA, USA). A substantial bias in the amino acid distribution was observed from positions −7 to +7 around the acetylated lysine residues. A total of 13 motifs were characterized in *A. japonicus* (Figure 2A). Among them, K^ac^XXK was the most common motif, followed by K^ac^L, KXXXXXXK^ac^, K^ac^F, and K^ac^Y (“K^ac^” represents the acetylated lysine, and “X” represents a random amino acid residue) (Figure 2B). The sequence logos showed that small hydrophobic residues were frequently around K^ac^, including leucine (L), valine (V), alanine (A), and glycine (G) (Figure 2C). Besides, lysine appears at a very high probability around K^ac^, especially from +3 to +7 and from −7 to −4 positions.

### 2.3. Functional Annotation of Acetylated Proteins

**Figure 3:**
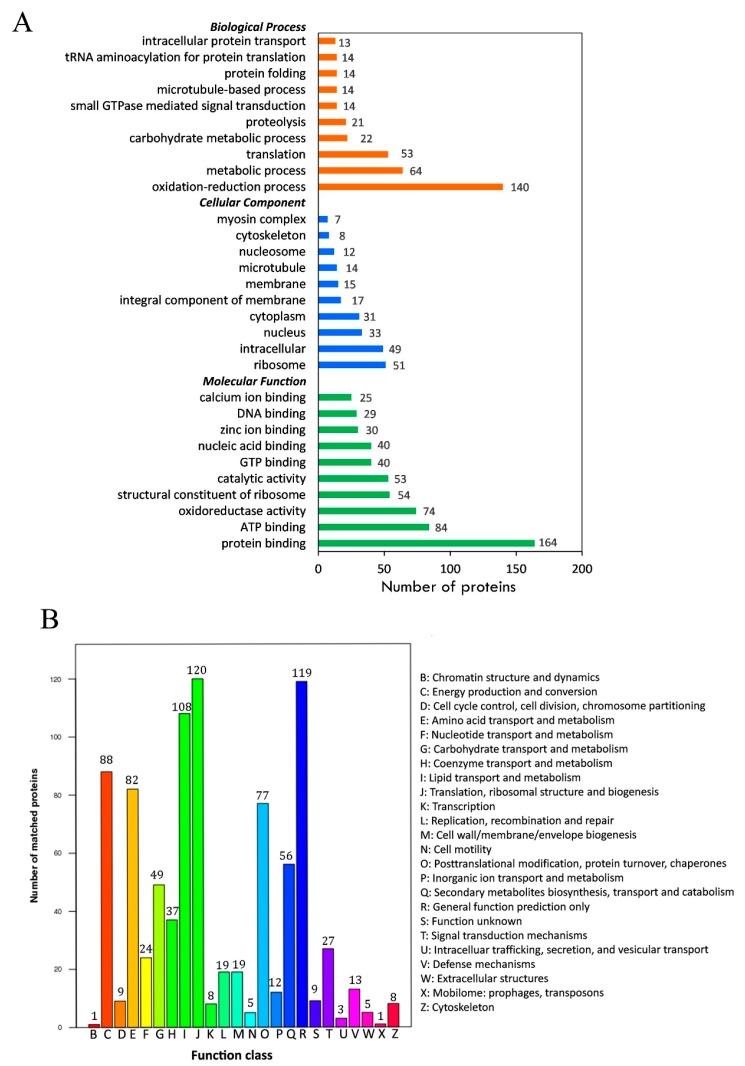
Functional classification of acetylated proteins identified in sea cucumbers. (**A**) Classification of acetylated proteins based on the Gene Ontology (GO) database. (**B**) Classification of acetylated proteins based on the Clusters of Orthologous Groups (COG) database.

The Gene Ontology (GO) analysis showed that a total of 951 acetylated proteins were annotated to GO terms. Three ontologies, including molecular function (MF), cellular component (CC), and biological process (BP), were further analyzed (Figure 3A). In the BP category, the major subcategories were “oxidation–reduction process” (140, 14.7%) and “metabolic process” (94, 9.9%). In the CC subcategory, a significant proportion of the clusters were classified as “ribosome” (51, 5.4%) and “intracellular” (40, 4.2%). In the MF subcategory, the largest subcategory was “protein binding” (164, 17.2%), followed by “ATP binding” (84, 8.8%) and oxidoreductase activity (74, 7.8%).

Clusters of Orthologous Groups (COG) annotation was also applied for the identified proteins. The results showed that the most abundant process was “translation, ribosomal structure, and biogenesis”, which was enriched with 120 proteins (Figure 3B). Both “lipid transport and metabolism” (with 108 proteins) and “post-translational modification, protein turnover, chaperones” with (77 proteins) were also of great abundance. To better describe the identified proteins, we also searched them in the Kyoto Encyclopedia of Genes and Genomes (KEGG) and the Interpro (IPR) database. The detailed information is listed in Table S1.

### 2.4. The Number of Acetylation Sites in Many Functional Proteins Changed Significantly under Heat Stress (HS)

**Figure 4:**
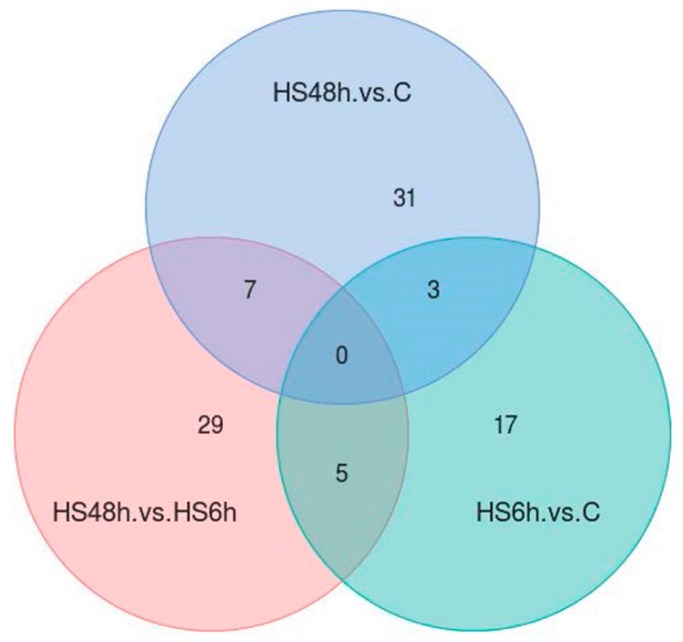
The number of acetylated sites with differential amounts among three comparisons. The criteria were *p*-value < 0.05 and fold changes (FCs) > 1.2 or < 0.83.

By the quantification of the acetylated sites, a total of 25, 41, and 41 differential quantified acetylated sites were found in these three comparisons respectively (Figure 4). The results also showed that there were some overlapping sites with differential acetylated quantitation among the three comparisons. Notable, 3 acetylated sites (K178 of translation initiation factor 2 subunit 1, K29 of N-terminal kinase-like protein, and K120 of spectrin beta) had different amounts in both HS6h versus C and HS48h versus C comparisons.

#### 2.4.1. HS6h versus C Comparison

A total of 25 differential modified acetylated sites were identified in the HS6h versus C comparison. The results showed that 20 acetylated sites were significantly up-regulated, while only 5 acetylated sites were significantly down-regulated. Three proteins with acetyltransferase activity, including histone acetyltransferase KAT5, KAT8 regulatory NSL complex subunit 1 (KANSL1), and CREB-binding protein (CBP), showed increased acetylation quantitation in some lysine sites in the 6 h group (Table 1). Two ribosomal proteins displayed up-regulated acetylation quantitation, including large subunit ribosomal protein L3e (Ribosomal_L3e) and small subunit ribosomal protein S15e (Ribosomal_S15e). Moreover, the acetylation quantitation of some translation-related proteins, such as translation initiation factor 2 (IF2), elongation factor 1α (EF1α), and elongation factor 2 (EF2), were up-regulated in the HS6h group compared the control group. Regarding the metabolic genes, the acetylation quantitation of K387 site in serine hydroxymethyltransferase (SHMT) decreased in the 6 h group. The acetylation amount of cholinesterase 1 (CHLE) was also down-regulated in the 6 h group.

#### 2.4.2. HS48h versus C Comparison

The changes in acetylation amount in HS48h was much more obvious, with 41 differential quantified acetylated sites compared with the control group (Table 2). Of these sites, 20 sites increased, while the acetylation amount of the other 21 sites declined.

Three lysine sites of CBP were involved in the changes: both K1583 and K1590 sites showed up-regulated acetylation quantitation, while the acetylation amount of K1766 was down-regulated. Besides, the K60 site of chromatin modification-related protein EAF6 exhibited a decreased acetylation amount.

Remarkably, various lysine sites of some chaperones were also involved in the different acetylation amounts. A total of 9 lysine sites (K147, K225, K316, K395, K440, K469, K476, K590, and K596) were identified in heat shock protein 90 (HSP90). Of these sites, K440 of HSP90 showed an increased acetylation amount in the HS48h group. T-complex protein 1 (TCP1), belonging to distant homologues of the HSP60 family, also showed an up-regulated acetylation amount in K206. On the contrary, the acetylation amounts of caseinolytic peptidase B protein (CLPB) and cyclophilin A (CYP1) were down-regulated in the HS48 group. The acetylation quantitation of the K87 site in glutathione S-transferase (GST) was up-regulated.

Ribosomal proteins showed different acetylation amounts in the HS48 group, but with opposite regulation of the HS6h group. Three lysine sites, respectively, in 60S ribosomal protein L23a (Ribosomal_L23a), large subunit ribosomal protein L3e (Ribosomal_L3e), and ribosomal protein S5 (Ribosomal_S5) showed declined acetylation amounts in the HS48 group. Besides, the acetylation amount of EF1α still increased, while the acetylation quantitation of EF2 decreased in the HS48 group.

Solute carrier family 39 member 10 (SLC39A10), also known as zinc transporter ZIP10, was found to show an increasing acetylation amount at the K43 site. Similarly, sodium-dependent organic anion transporter (NPT) had a higher acetylation amount at K114 in the HS48h group.

#### 2.4.3. HS48h versus HS6h Comparison

There were 41 lysine sites (21 up-regulated and 20 down-regulated) that had different acetylation amounts in the HS48h versus HS6h comparison. The results in this comparison shared some acetylated sites with those in the above two comparisons. For example, HSP60, FANCJ, EF1α, and some ribosome proteins were still included (Table 3). It is worth noting that various proteins involved in ion transport regulated the acetylation amount significantly in this comparison. For example, diverse proteins in solute carrier families (SLCs), such as NPT, SLC39A10, SLC25 and SLC28, increased the acetylation quantitation, while the acetylation amount of proteins in the transferrin family declined.

### 2.5. Differentially Acetylated Proteins Represented Specific Interaction Networks

For the purpose of understanding how these acetylated proteins interacted with each other, we used the STRING database (https://string-db.org) and Cytoscape software to construct the protein–protein interaction (PPI) networks for the proteins with differentially acetylated sites. The results showed that 37 proteins were mapped to the protein interaction database. These proteins were clustered into one big cluster and 5 protein pairs (Figure 4).

**Figure 5:**
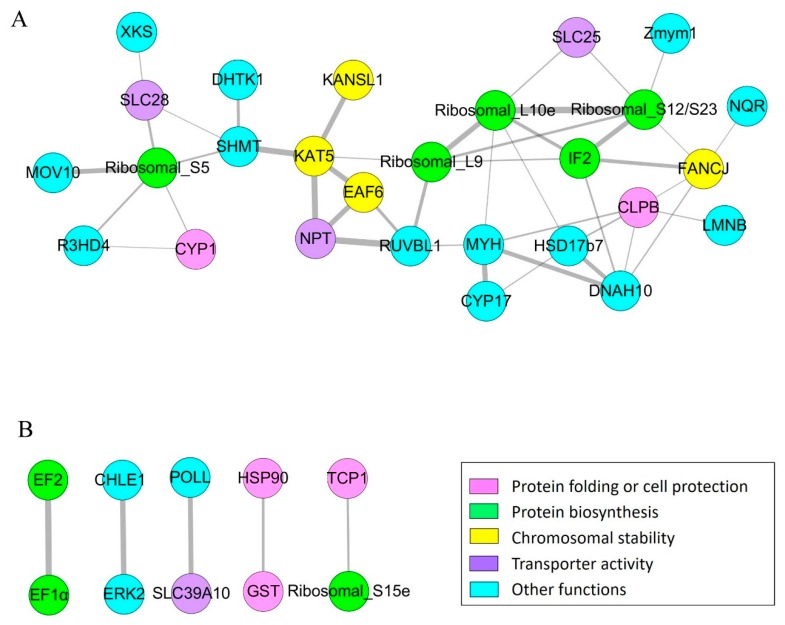
Interaction networks of the proteins with differential numbers of acetylated sites. (**A**) The major interaction network of the proteins with differential numbers of acetylated sites. (**B**) Five proteins pairs with interactions. The color of the circles depends on the functions of the proteins. The width of the lines is positively correlated with the strength of interaction.

The top group consisted of 27 proteins, including some ribosomal proteins, translation-associated proteins, and proteins associated with chromosomal stability (Figure 5A). Remarkably, translation initiation factor 2 (IF2), as one of the center proteins, had strong interactions with a variety of proteins, including ribosomal protein S12/S23 (Ribosomal_S12/S23), large subunit ribosomal protein L10e (Ribosomal_L10e), 39S ribosomal protein L9 (Ribosomal_L9), Fanconi anemia group J protein homolog (FANCJ), and dynein heavy chain 10 (DNAH10). Additionally, histone acetyltransferase KAT5, ribosomal protein S5 (Ribosomal protein_S5), and myosin (MYH) were key proteins, which had strong connections with some other proteins.

There were 5 protein pairs with interactions (Figure 5B). Results implied that elongation factor 2 (EF2) and translation elongation factor EF-1 alpha (EF1α) closely interacted with each other. Besides, HSP90 and GST, HSP60 and Ribosomal_S15e, cholinesterase 1 (CHLE1) and extracellular signal-regulated kinase 2 (ERK2), and pol-like protein (POLL) and SLC39A10 closely interacted with each other, respectively.

## 3. Discussion

### 3.1. Lysine Acetylation Profiles in A. japonicus

In this study, we used global mass spectrometry based acetylproteome profiling to identify 4028 lysine acetylation sites arising from 1439 proteins in the intestine tissue of *A. japonicus*. A total of 13 motifs were characterized. K^ac^XXK was the most common motif, followed by K^ac^L, KXXXXXXK^ac^, K^ac^F, and K^ac^Y. Of these motifs, K^ac^XXK, K^ac^F, and K^ac^Y were reported in mammals and bacteria, which indicated extensively conserved sequences of these motifs [22,23,24]. Sequence logos showed that lysine was over-represented immediately around acetylated lysine in *A. japonicus*. The preference was also observed in many other species [25,26]. These motifs may be specially recognized by diverse lysine acetyltransferases (KATs). Further studies are needed to identify the corresponding KATs that recognize these acetylation motifs.

By GO functional analysis of the acetylated proteins, we found “protein binding” was one of the most abundant terms in the MF category in *A. japonicus*, which was consistent with the findings in bacteria [23], plants [25], and mammals [24]. The terms associated with “ribosome”, “nucleus”, “cytoplasm”, and “membrane” were enriched in the CC category, which suggested the wide distribution of lysine acetylated proteins [25]. The acetylated proteins we identified also represented a wide range of BP terms. The result of COG analysis also implied that ribosomal proteins exhibited wide acetylation in *A. japonicus*, as reported in other species [25,27]. Besides, acetylation targeted proteins involved in “post-translational modification, protein turnover, chaperones” and “lipid transport and metabolism”. These proteins had broad, important regulatory roles in HSR [28]. Overall, the functional analysis implied that lysine acetylation existed in proteins of *A. japonicus* widely, including the stress response proteins.

### 3.2. Differential Number of Acetylated Sites under HS

We studied the expression of protein in our previous proteomic study [28]. In that study, a total of 3432 proteins were identified, and 127 proteins showed significant difference in the C versus HS48h comparison. In the present study, 4028 lysine acetylation sites in 1439 proteins were identified by acetylproteome sequencing, and 41 acetylation sites had differential amounts in the C versus HS48h comparison. We summarized these data in the two studies, and found that only five proteins were significantly changed in both protein and acetylation amounts. The five proteins included heat shock protein 90 (HSP90), ELL-associated factor 2-like (EAF2), extracellular signal-regulated kinase 2 (ERK2), gamma-butyrobetaine dioxygenase (BBOX), and phosphodiesterase family member 7-like (ENPP7), and the changes in protein and acetylation amounts are listed in Table S2. Thus, we suppose that the quantitative changes of most acetylated sites resulted from protein modification rather than from protein abundance. The differential amounts of acetylated sites generally reflected the acetylation levels of these sites [24,27].

#### 3.2.1. Lysine Acetyltransferases (KATs) and Deacetylases (KDACs)

KATs and KDACs are often referred to as histone acetyltransferases (HATs) and histone deacetylases (HDACs), respectively, because lysine acetylation was initially identified in histones [7]. Genome-wide sequence analyses in *A. japonicus* have indicated the presence of diverse KATs, such as MYST proteins and CREB-binding protein (CBP), as well as diverse kinds of KDACs [29].

Many KATs and KDACs themselves are autoacetylated [7]. Prominent examples are CBP and its homologue p300. CBP and p300 proteins are transcriptional co-activators of various transcription factors that are involved in a wide array of cellular activities, such as DNA repair, cell growth, differentiation, and apoptosis [30,31,32]. A reduced catalytic activity of p300 was achieved by autoacetylation of a cluster of acetylated sites within an apparent activation loop motif [33]. In the present study, we found that CBP (NCBI sequence ID: PIK57779.1) possessed conserved modular domains with the protein in other species. Notably, four lysine sites with close locations (K1583, K1590, K1766, and K1804) had different acetylation amounts under HS. These four lysine sites were around the zinc-binding sites as well as hydrophobic binding surface (Figure S1). The three-dimensional domain structures of partial CBP predicted by the SWISS-MODEL server also proved that these lysine residues were very close to zinc ion binding sites, especially K1804. We, therefore, proposed that these acetylated lysine sites could alter the activities and functions of CBP and then influence a wide variety of signaling and transcriptional events under HS [33].

#### 3.2.2. Chaperones

In a previous report in Hela cells, lysine acetylation was identified in chaperone proteins, including HSP70, HSP27, HSP90, TCP1(T-complex protein 1), and cyclophilin A (CYP1) [34]. In *A. japonicus*, we found many chaperones could be lysine acetylated, including HSP70, HSP90, HSP110, and HSP60 families, as well as small HSPs (HSP26) and CYP1. Of these chaperones, HSP90, CLBP (HSP78), TCP1 (distant homologues of HSP60 family), and CYP1 had distinct acetylation quantitation under HS.

HSP90 is critical for the maturation of many crucial transcription factors, and acetylation regulation of HSP90 has drawn the most attention among chaperons. HSP90 interacts with transcription factor glucocorticoid receptor (GR), which helps GR to achieve a conformation suitable for binding its ligand and subsequent translocation into the nucleus. Acetylation of HSP90 prevents this interaction, ultimately leading to a loss of transcriptional activity of GR [35]. HDAC6, which binds and deacetylates HSP90, has been identified as a regulator of HSP90 acetylation and its chaperone activity [36,37,38]. A conserved HSP90 residue (K294), which is located in the middle region and C-terminal from the charged linker, is regarded as a key regulation site for HSP90 [7]. In yeast, K294 acetylation weakens the association with co-chaperones and client proteins [39]. In *A. japonicus* HSP90, K327 is equivalent to K294 in the human protein. Surprisingly, K327 was not an acetylation site in our data. In our study, we identified a total of nine lysine sites (K147, K225, K316, K395, K440, K469, K476, K590, and K596) in HSP90, which proved the presence of multiple acetylation sites in HSP90. Under HS48h, only the acetylation amount of K440 was up-regulated, to 1.94-fold. From our previous result, the protein expression of HSP90 rose dramatically in the HS48h group, by a factor of 6.11 [28]. Hence, we suppose the acetylation level of HSP90 (K440), which was represented with a ratio of the number of acetylated sites and amount of protein expression, was also reduced under HS in *A. japonicus* [35,39]. The results also showed that the regulation of HSP90 in *A. japonicus* under HS occurred at both translation and PTM levels. The specific characteristics and regulation mechanism in the acetylation of HSP90 still needs exploring.

CYP1, a peptidyl-prolyl isomerase, binds the immunosuppressive drug cyclosporin A and modulates T cell activation by inhibiting the phosphatase calcineurin [40]. Disruption of CYP1 decreases survival of cells following exposure to high temperatures, indicating that CYP1 plays a role in the stress response [41]. CYP1 is acetylated at K125, a residue located in the loop surrounding the drug pocket and a potential site of ionic interaction with calcineurin [34]. Acetylation of K125 could affect the interaction of cyclophilin with calcineurin. In our present study, CYP1 had decreasing acetylation amounts at both K47 and K80 sites in the HS48h group compared with the control group. No significant changes in the protein expression of CYP1 were examined in HSR of *A. japonicus*. We speculated that the deacetylation of CYP1 contributed to its isomerase activity under HS.

#### 3.2.3. Translation-Associated Factors and Ribosome Proteins

Our results showed that the acetylation amounts of three translation-related factors (IF2, EF1, and EF2) increased significantly under HS in *A. japonicus*. Previous reports proved that lysine acetylation occurred in diverse EFs [42,43]. However, rare evidence is obtained about the impact of acetylation on the activity of these translation factors. Besides, some ribosome proteins had differential acetylation amounts in this study. Generally, the acetylation amounts of Ribosomal_L3e and Ribosomal_S15e increased at HS6h, while those of Ribosomal_L23e and Ribosomal_S5 declined at HS48h. Modifications of ribosomal proteins are important for protein synthesis [44]. For example, phosphorylation of ribosomal protein S6 has key regulatory roles in cell size and glucose homeostasis [45]. The acetylation level of mitochondrial ribosomal protein L10 influences mitochondrial protein synthesis [46]. We supposed that the dynamic acetylation changes of translation-related factors and ribosome proteins acted as the complex regulation mode on the protein synthesis under HS.

## 4. Materials and Methods

### 4.1. Samples

*A. japonicus* (100–120 g) was collected from an aquaculture farm in Weihai (Shandong, China) in April 2018. The sea cucumbers were transported to the tanks in our lab of Qingdao. The temperature and the salinity of sea water were around 16 °C and 30‰, respectively, before the treatment experiment started. Acclimation in tanks lasted for 2 weeks. During both the acclimation and treatment experiment, the sea cucumbers were fed with regular fodder, and half of the water was changed daily [47].

When acclimation finished, three individuals taken from the tank were regarded as the control group. The methods of heating and sampling were followed by our previous procedures. Briefly, a heating rod with 2 kW of power was placed in the tank. The rate of heating was about 2 °C/h. The heating temperature was set to 26 °C, and then water temperature was maintained at 26 ± 0.5 °C during the subsequent experiment. The initial time was considered when the temperature just reached 26 °C. Three individuals were taken randomly after 6 and 48 h stimulations. The intestine tissue of these individuals was tweezed and washed with ddH_2_O, which helped us clear the intestine contents as much as possible. All samples were frozen quickly in liquid nitrogen and then stored at –80 °C.

### 4.2. Protein Extraction

The sample was ground with pestles in liquid nitrogen, respectively, and the sample powder was mixed with extraction buffer containing 8 M urea, 1% Triton-100, 65 mM dithiothreitol (DTT), 0.1% protease inhibitor cocktail, 3 μM trichostatin A, and 50 mM nicotinamide. Then, the sample was sonicated three times on ice using a high-intensity ultrasonic processor. After centrifugation at 20,000× *g* at 4 °C for 10 min, the protein in the supernatant was precipitated with 15% cold trichloroacetic acid (TCA) at −20 °C for 2 h. The protein was washed with cold acetone three times. Finally, the protein was re-dissolved in buffer (8 M urea, 100 mM NH_4_HCO_3_, pH 8.0), and the protein concentration was determined by a bicinchoninic acid (BCA) quantitative kit (Beyotime, Shanghai, China).

### 4.3. Trypsin Digestion and ENRICHMENT of Lysine-Acetylated Peptides

A total of 10 mg protein was reduced with 10 mM DTT for 1 h at 37 °C and alkylated with 20 mM iodoacetamide for 30 min at room temperature in darkness. For trypsin digestion, the protein sample was diluted in 0.1 M triethylammonium bicarbonate (TEAB). Finally, the diluted protein samples were digested with trypsin at 1:50 trypsin-to-protein mass ratio for 12 h first, and then 1:100 trypsin-to-protein mass ratio for 4 h.

To enrich acetylated peptides, the tryptic peptides were dissolved in NETN buffer (100 mM NaCl, 1 mM EDTA, 50 mM Tris-HCl, 0.5% NP-40, pH 8.0) and incubated with anti-acetyl lysine antibody beads (PTM Biolabs, Hangzhou, China) at 4 °C for 12 h with gentle shaking. The beads were washed four times with NETN buffer and twice with ddH_2_O. The bound peptides were eluted with 0.1% trifluoroacetic acid (TFA). In preparation for analysis, the peptides were desalted using peptide desalting spin columns (ThermoFisher, Waltham, MA, USA).

### 4.4. Liquid Chromatography–Tandem Mass Spectrometry (LC-MS/MS) Detection and Data Analysis

The eluted peptides were cleaned with C18 ZipTips (Millipore, Bedford, MA, USA) according to the manufacturer’s instructions. LC-MS/MS analyses were performed according to the previous report [48]. Briefly, tryptic peptides were dissolved in solvent A (0.1% formic acid) and loaded onto a homemade reversed-phase analytical column (15 cm length, 75 µm inside diameter). Solvent B was 0.1% formic acid in 80% acetonitrile. The gradient elutions were carried out at a constant flow rate of 400 nL/min on an EASY-nLC 1000 UPLC system. The peptides were subjected to an NSI source followed by MS/MS analysis, which was performed in EASY-nLC 1200 UHPLC (Thermo Fisher Scientific, Madison, WI, USA) coupled to an Orbitrap Q Exactive HF-X mass spectrometer (Thermo Fisher Scientific, Madison, WI, USA) operating in the data-dependent acquisition (DDA) mode. For data-dependent analysis (DDA), a Q-Exactive HF-X mass spectrometer was operated in positive polarity mode with a spray voltage of 2.3 kV and a capillary temperature of 320 °C. Full MS scans from 350 to 1500 m/z were acquired at a resolution of 60,000 (at 200 m/z) with an automatic gain control (AGC) target value of 3 × 10^6^ and a maximum ion injection time of 20 ms. From the full MS scan, a maximum number of 40 of the most abundant precursor ions were selected for higher energy collisional dissociation (HCD) fragment analysis at a resolution of 15,000 (at 200 m/z) with an AGC target value of 1 × 10^5^, a maximum ion injection time of 45 ms, a normalized collision energy of 27%, and an intensity threshold of 8.3 × 10^3^, and the dynamic exclusion parameter was set at 60 s. The MS spectra were collected in the 350–1500 m/z range.

The MS/MS data were processed using Proteome Discoverer 2.2 software (PD2.2, Waltham, MA, USA), and the processed data were searched against the *A. japonicus* genome database concatenated with a reverse decoy database [29]. The identification parameters were set as follows: precursor mass tolerance of 10 ppm and fragment mass tolerance of 0.02 Da. Trypsin/P was specified as the cleavage enzyme, allowing up to two missed cleavages, five modifications per peptide, and five charges. Carbamidomethylation on Cys was specified as a fixed modification, and oxidation on Met, phosphorylation on Ser, Thr, Tyr, and acetylation on the protein N-terminus were specified as variable modifications [49]. False discovery rate thresholds for peptide, protein, and modification site were specified at 5%. Minimum peptide length was set at seven residues. The site localization probability was set as >0.5. All other parameters in PD 2.2 software were set to default values. We checked the mass error of all identified peptides; the distribution was close to zero, and most were less than 0.02 Da, confirming that the mass accuracy of the MS data was acceptable.

The raw quantification values were calculated and validated using the Minora Feature Detector node in the Processing workflow of PD2.2. A quantification value was the intensity, and the area was detected for a given quantification channel depending on the setting in the precursor ions quantifier in the consensus method. A t-test with a *p*-value < 0.05 was applied to distinguish the differential quantitation of acetylated sites [26,49,50]. Fold changes (FCs) >1.2 or <0.83 were also regarded as the criteria of differentially acetylated sites [51]. Data are available via ProteomeXchange with the dataset identifier PXD013929.

### 4.5. Bioinformatics Analysis

Software motif-x (Boston, MA, USA) was used to analyze the model of sequences constituted with amino acids in specific positions (7 amino acids upstream and downstream of the acetylation site) in all acetylated proteins identified. Function annotations, such as GO and KEGG analyses, were performed as previously described [46]. COG annotation and IPR annotation were also performed to better classify the functions of proteins [52]. PPI for the identified differentially acetylated proteins were performed using the STRING database and Cytoscape software.

The amino acid sequence of CREB-binding protein (CBP) was analyzed for similarity with the BLAST programs at the National Center for Biotechnology Information (http://www.ncbi.nlm.nih.gov/BLAST/). The three-dimensional domain structure of partial CBP was predicted by the SWISS-MODEL server (https://swissmodel.expasy.org/interactive).

## 5. Conclusions

In summary, this study presents a comprehensive analysis of acetylproteome in the sea cucumber *A. japonicus*. The profiling of acetylation proteins involved in HSR was identified in the species. Moreover, key proteins, such as lysine acetyltransferases, chaperones, translation-associated factors, and ribosome proteins, showed differential acetylation amounts under HS, suggesting complex post-translational modification was involved in the process. Collectively, our results offer novel insight into HSR in *A. japonicus* and provide a resource for further mechanistic studies examining the regulation of protein functions by lysine acetylation.

## Figures and Tables

**Table 1:** Proteins with differential acetylation amounts in the HS6h vs. control (C) comparison.

Accession No.	Function	Protein Name	Short Name	Acetylated Site	log_2_value
AJAP27758	KAT KADC	histone acetyltransferase KAT5	KAT5	K124↑	0.86
AJAP07562	KAT KADC	KAT8 regulatory NSL complex subunit 1	KANSL1	K672↑	0.62
AJAP05305	KAT KADC	CREB-binding protein	CBP	K1804↑	0.46
AJAP01782	chromosomal stability	Fanconi anemia group J protein homolog	FANCJ	K274↑	1.28
AJAP09062	ribosome	large subunit ribosomal protein L3e	Ribosomal_L3e	K456↑	1.19
AJAP03812	ribosome	small subunit ribosomal protein S15e	Ribosomal_S15e	K78↑	1.00
AJAP22222	protein biosynthesis	translation initiation factor 2 subunit 1	IF2	K178↑	1.03
AJAP07581	protein biosynthesis	translation elongation factor 2	EF2	K19↑	0.67
AJAP06040	protein biosynthesis	translation elongation factor EF-1alpha (GTPase)	EF1α	K320↑	1.57
AJAP00690	protein binding	bromodomain adjacent to zinc finger domain protein 1B	BAZ1B	K905↑	0.65
AJAP15703	protein binding and apoptosis	peptidyl-tRNA hydrolase 2, mitochondrial	PTH2	K128↑	0.93
AJAP23458	protein binding	dynein heavy chain 10, axonemal isoform X3	DNAH10	K909↑	0.82
AJAP04502	DNA binding	N-terminal kinase-like protein	NTKL	K29↑	0.86
AJAP17355	transport activity	melanotransferrin	MELTF	K85↑	1.40
AJAP15405	glycolytic process	acyl-CoA dehydrogenase family member 10	ACAD10	K416↑	0.62
AJAP21096	threonine catabolic	glycine C-acetyltransferase	GCAT	K97↑	1.30
AJAP15667	oxidation-reduction	NADPH:quinone reductase or related Zn-dependent oxidoreductase	NQR	K56↑	0.72
AJAP10667	xylulose catabolic	xylulokinase	XKS	K359↑	1.82
AJAP24097	DNA integration	Retrovirus-related Pol polyprotein from transposon/ Retro element 1	RE1	K30↑	1.26
AJAP20997	tRNA modification	queuine tRNA-ribosyltransferase-like	TGTL	K49↑	0.51
AJAP07945	protein binding	spectrin beta	Spectrinβ	K120↓	−0.70
AJAP19047	oxidation-reduction	2-oxoglutarate dehydrogenase E1 component DHKTD1	DHKTD1	K573↓	−1.21
AJAP05715	transferase activity	Serine hydroxymethyltransferase	SHMT	K387↓	−1.21
AJAP19958	hydrolysis activity	cholinesterase 1	CHLE1	K63↓	−0.41
AJAP17228	--	hypothetical protein	--	K1127↓	−0.76

**Table 2:** Proteins with differential acetylation amounts in the HS48h vs. C comparison.

Accession No.	Function	Protein Name	Short Name	Acetylated Site	log_2_value
AJAP05305	KAT KADC	CREB-binding protein	CBP	K1583↑	0.64
AJAP05305	KAT KADC	CREB-binding protein	CBP	K1590↑	0.64
AJAP05305	KAT KADC	CREB-binding protein	CBP	K1766↓	−1.28
AJAP23933	KAT KADC	chromatin modification-related protein Eaf6	EAF6	K60↓	−0.96
AJAP26676	protein folding	heat shock protein 90	HSP90	K440↑	0.96
AJAP22974	protein folding	T-complex protein 1	TCP1	K206↑	0.68
AJAP29146	protein folding	caseinolytic peptidase B protein homolog (HSP78)	CLPB	K576↓	−1.38
AJAP27049	protein folding	cyclophilin A (peptidyl-prolyl cis-trans isomerase)	CYP1	K47↓	−1.28
AJAP27049	protein folding	cyclophilin A (peptidyl-prolyl cis-trans isomerase)	CYP1	K80↓	−0.69
AJAP04150	cell protection	glutathione S-transferase	GST	K87↑	0.51
AJAP22222	protein biosynthesis	translation initiation factor 2 subunit 1	IF2	K178↑	2.04
AJAP06040	protein biosynthesis	translation elongation factor EF-1alpha (GTPase)	EF1α	K338↓	−1.12
AJAP15989	transporter activity	solute carrier family 39 member 10 (zinc transporter ZIP10-like)	SLC39A10	K43↑	1.20
AJAP16427	transporter activity	sodium-dependent organic anion transporter	NPT	K114↑	0.59
AJAP03428	apoptosis	ELL-associated factor 2-like	EAF2	K259↑	1.13
AJAP01277	apoptosis	MAP kinase-activating death domain protein-like	MADD	K419↓	−0.66
AJAP20633	oxidation-reduction	2-hydroxyglutarate dehydrogenase	2HDH	K393↑	0.62
AJAP22233	oxidation-reduction	gamma-butyrobetaine dioxygenase	BBOX	K479↓	−2.72
AJAP21026	cytoskeletal binding	lamin B	LMNB	K51↑	1.10
AJAP27911	cytoskeletal binding	tropomyosin	TPM	K365↑	1.04
AJAP09828	cytoskeletal binding	myosin	MYH	K31↓	−0.63
AJAP21063	regulation of cytoskeleton	bromodomain and WD repeat-containing protein 3-like, partial	BRWD3	K81↑	1.09
AJAP04260	DNA integration	transposon Ty3-I Gag-Pol polyprotein	TY3B-I	K272↑	0.63
AJAP04502	retrograde vesicle-mediated transport	N-terminal kinase-like protein	NTKL	K29↑	0.97
AJAP20141	cofactor binding	cytohesin-3-like, partial	CYTH3	K569↑	1.27
AJAP28079	glycolytic process	fructose-bisphosphate aldolase, class I	FBA1	K219↑	0.65
AJAP00341	metabolic process	extracellular signal-regulated kinase 2	ERK2	K220↑	1.24
AJAP12117	amino acid metabolism	branched-chain-amino-acid aminotransferase-like protein 1	BCAT1	K746↑	1.08
AJAP17627	ATP binding	multidrug resistance-associated protein 5	MRP5	K51↑	0.81
AJAP13674	ribosome	60S ribosomal protein L23a	Ribosomal_L23a	K59↓	−1.12
AJAP09062	ribosome	large subunit ribosomal protein L3e	Ribosomal_L23e	K225↓	−1.35
AJAP23675	ribosome	Ribosomal protein S5	Ribosomal_S5	K115↓	−1.17
AJAP04718	endoplasmic reticulum membrane	putative helicase MOV-10	MOV-10	K349↓	−0.81
AJAP24952	transaminase activity	SH2 domain-containing protein 4a-like, partial	SH2D4	K313↓	−1.41
AJAP05049	DNA helicase activity	RuvB-like protein 1 (pontin 52) DNA helicase TIP49, TBP-interacting protein	RUVBL1	K453↓	−0.60
AJAP07945	protein binding	spectrin beta	SPTB	K120↓	−0.46
AJAP10927	protein binding	phosphodiesterase family member 7-like	ENPP7	K840↓	−1.01
AJAP26499	nucleic acid binding	RNA recognition motif (RRM) domain	RRM	K101↓	−0.74
AJAP27984	nucleic acid binding	R3H domain-containing protein 4	R3HD4	K79↓	−1.00
AJAP07965	--	hypothetical protein	--	K933↓	−0.56
AJAP24653	--	hypothetical protein	--	K916↓	−0.88

**Table 3:** Proteins with differential acetylation amounts in the HS48h vs. HS6h comparison.

Accession No.	Function	Protein Name	Short Name	Acetylated Site	log_2_value
AJAP22974	protein folding	T-complex protein 1	TCP1	K206↑	0.93
AJAP01782	chromosomal stability	Fanconi anemia group J protein homolog	FANCJ	K274↓	−0.50
AJAP06040	protein biosynthesis	translation elongation factor EF-1alpha (GTPase)	EF1α	K192↑	0.91
AJAP16427	transporter activity	sodium-dependent organic anion transporter	NPT	K114↑	1.01
AJAP03608	transporter activity	solute carrier family 25 (mitochondrial adenine nucleotide translocator)	SLC25	K95↑	0.44
AJAP15989	transporter activity	solute carrier family 39 member 10 (zinc transporter ZIP10-like)	SLC39A10	K43↑	1.06
AJAP17787	transporter activity	solute carrier family 28 member 3-like	SLC28	K370↑	0.29
AJAP09671	transporter activity	cyclic nucleotide-gated cation channel alpha-3-like	CNGA3	K669↑	0.97
AJAP09671	transporter activity	cyclic nucleotide-gated cation channel alpha-3-like	CNGA3	K671↑	0.97
AJAP29476	transporter activity	zinc finger BED domain-containing protein 4-like	Zbed4	K117↑	0.73
AJAP13059	transporter activity	zinc finger MYM-type protein 1-like	Zmym1	K119↓	−1.10
AJAP17356	transporter activity	transferrin-like domain	TFD	K42↓	−2.03
AJAP17356	transporter activity	transferrin-like domain	TFD	K674↓	−1.54
AJAP17355	transporter activity	melanotransferrin	MELTF	K85↓	−1.60
AJAP18698	oxidation-reduction	Thiol-disulfide isomerase/thioredoxin	TRX	K51↑	0.97
AJAP20633	oxidation-reduction	2-hydroxyglutarate dehydrogenase	2HDH	K393↑	0.77
AJAP22233	oxidation-reduction	gamma-butyrobetaine dioxygenase	BBOX	K479↓	−2.58
AJAP15456	autophagy	autophagy-related protein 18	ATG18	K203↑	0.80
AJAP12353	apoptosis	ADAMTS-like protein 2 isoform X1	Adamtsl2	K621↓	−0.41
AJAP01277	apoptosis	MAP kinase-activating death domain protein-like	MADD	K419↓	−0.87
AJAP28079	protein binding	fructose-bisphosphate aldolase, class I	ALDOA	K60↑	0.95
AJAP11248	steroid metabolic	steroid 17-alpha-hydroxylase/17,20 lyase	CYP17	K75↑	1.24
AJAP09155	cholesterol biosynthetic	3-keto-steroid reductase/17-beta-hydroxysteroid dehydrogenase 7	HSD17b7	K200↑	0.92
AJAP19958	hydrolysis activity	cholinesterase 1	CHLE1	K63↑	0.60
AJAP19958	hydrolysis activity	cholinesterase 1	CHLE1	K191↓	−0.97
AJAP27911	cytoskeletal protein binding	tropomyosin	TPM	K365↑	1.07
AJAP14831	hydrolase activity	acyl-coenzyme A thioesterase 9	ACOT9	K93↑	1.11
AJAP15858	ribosome	39S ribosomal protein L9, mitochondrial isoform X3	Ribosomal_L9	K191↑	1.17
AJAP17052	ribosome	large subunit ribosomal protein L10e	Ribosomal_L10e	K101↓	−0.76
AJAP05814	ribosome	ribosomal protein S12/S23	Ribosomal_S12/S23	K44↓	−0.62
AJAP15405	glycolytic process	acyl-CoA dehydrogenase family member 10	ACAD10	K416↓	−0.67
AJAP21933	glycolytic process	pyruvate kinase	PKA	K100↓	−0.62
AJAP29031	negative regulation of translation	GRB10-interacting GYF protein 2	GIGYF2	K147↓	−1.29
AJAP15703	protein binding	peptidyl-tRNA hydrolase 2, mitochondrial	PTH2	K128↓	−1.48
AJAP07321	metal binding	serum paraoxonase/arylesterase 1-like	PON	K201↓	−1.62
AJAP26236	metal binding	pol-like protein	POLL	K338↓	−0.57
AJAP08618	GTPase activation	Rab GDP dissociation inhibitor	GDI	K147↓	−0.55
AJAP09462	G-protein coupled receptor	alpha-1A adrenergic receptor	ADA1A	K123↓	−1.10
AJAP16917	--	hypothetical protein	--	K345↓	−1.59
AJAP16076	--	hypothetical protein	--	K131↑	1.19
AJAP05930	--	hypothetical protein	--	K174↑	1.44

## Supplementary Materials

Supplementary materials can be found at https://www.mdpi.com/1422-0067/20/18/4423/s1.

## References

[1] Hashiguchi A., Komatsu S. (2016). Impact of Post-Translational Modifications of Crop Proteins under Abiotic Stress. Proteomes.

[2] Seet B.T., Dikic I., Zhou M.-M., Pawson T. (2006). Reading protein modifications with interaction domains. Nat. Rev. Mol. Cell Biol..

[3] Allfrey V., Faulkner R., Mirsky A. (1964). Acetylation and methylation of histones and their possible role in the regulation of RNA synthesis. Proc. Natl. Acad. Sci. USA.

[4] Grunstein M. (1997). Histone acetylation in chromatin structure and transcription. Nature.

[5] Turner B.M. (2000). Histone acetylation and an epigenetic code. Bioessays.

[6] Glozak M.A., Sengupta N., Zhang X., Seto E. (2005). Acetylation and deacetylation of non-histone proteins. Gene.

[7] Yang X.-J., Seto E. (2008). Lysine acetylation: Codified crosstalk with other posttranslational modifications. Mol. Cell.

[8] Zhang Y., Song L., Liang W., Mu P., Wang S., Lin Q. (2016). Comprehensive profiling of lysine acetylproteome analysis reveals diverse functions of lysine acetylation in common wheat. Sci. Rep..

[9] Tauber C.A., Tauber M.J. (1981). Insect seasonal cycles: Genetics and evolution. Annu. Rev. Ecol. Syst..

[10] Hentchel K.L., Escalante-Semerena J.C. (2015). Acylation of biomolecules in prokaryotes: A widespread strategy for the control of biological function and metabolic stress. Microbiol. Mol. Biol. Rev..

[11] Neilson K.A., Gammulla C.G., Mirzaei M., Imin N., Haynes P.A. (2010). Proteomic analysis of temperature stress in plants. Proteomics.

[12] Liu F., Yang M., Wang X., Yang S., Gu J., Zhou J., Zhang X.-E., Deng J., Ge F. (2014). Acetylome analysis reveals diverse functions of lysine acetylation in *Mycobacterium tuberculosis*. Mol. Cell. Proteom..

[13] Ma Q., Wood T.K. (2011). Protein acetylation in prokaryotes increases stress resistance. Biochem. Biophys. Res. Commun..

[14] Westerheide S.D., Anckar J., Stevens S.M., Sistonen L., Morimoto R.I. (2009). Stress-inducible regulation of heat shock factor 1 by the deacetylase SIRT1. Science.

[15] Ozden O., Park S.-H., Kim H.-S., Jiang H., Coleman M.C., Spitz D.R., Gius D. (2011). Acetylation of MnSOD directs enzymatic activity responding to cellular nutrient status or oxidative stress. Aging.

[16] Du H., Bao Z., Hou R., Wang S., Su H., Yan J., Tian M., Li Y., Wei W., Lu W. (2012). Transcriptome Sequencing and Characterization for the Sea Cucumber *Apostichopus japonicus* (Selenka, 1867). PLoS ONE.

[17] Hoegh-Guldberg O., Bruno J.F. (2010). The impact of climate change on the world’s marine ecosystems. Science.

[18] Li C., Fang H., Xu D. (2019). Effect of seasonal high temperature on the immune response in *Apostichopus japonicus* by transcriptome analysis. Fish Shellfish Immunol..

[19] Xu D., Sun L., Liu S., Zhang L., Ru X., Zhao Y., Yang H. (2014). Molecular cloning of heat shock protein 10 (Hsp10) and 60 (Hsp60) cDNAs and their expression analysis under thermal stress in the sea cucumber *Apostichopus japonicus*. Comp. Biochem. Physiol. B Biochem. Mol. Biol..

[20] Zhao H., Yang H., Zhao H., Chen M., Wang T. (2011). The molecular characterization and expression of heat shock protein 90 (Hsp90) and 26 (Hsp26) cDNAs in sea cucumber (*Apostichopus japonicus*). Cell Stress Chaperones.

[21] Xu D., Zhou S., Sun L. (2018). RNA-seq based transcriptional analysis reveals dynamic genes expression profiles and immune-associated regulation under heat stress in *Apostichopus japonicus*. Fish Shellfish Immunol..

[22] Schwartz D., Chou M.F., Church G.M. (2009). Predicting Protein Post-translational Modifications Using Meta-analysis of Proteome Scale Data Sets. Mol. Cell Proteom..

[23] Huang D., Li Z.-H., You D., Zhou Y., Ye B.-C. (2015). Lysine acetylproteome analysis suggests its roles in primary and secondary metabolism in *Saccharopolyspora erythraea*. Appl. Microbiol. Biotechnol..

[24] Xie C., Shen H., Zhang H., Yan J., Liu Y., Yao F., Wang X., Cheng Z., Tang T.-S., Guo C. (2018). Quantitative proteomics analysis reveals alterations of lysine acetylation in mouse testis in response to heat shock and X-ray exposure. BBA Proteins Proteom..

[25] Jiang J., Gai Z., Wang Y., Fan K., Sun L., Wang H., Ding Z. (2018). Comprehensive proteome analyses of lysine acetylation in tea leaves by sensing nitrogen nutrition. BMC Genom..

[26] Zhu G.-R., Yan X., Zhu D., Deng X., Wu J.-S., Xia J., Yan Y.-M. (2018). Lysine acetylproteome profiling under water deficit reveals key acetylated proteins involved in wheat grain development and starch biosynthesis. J. Proteom..

[27] Zhou Z., Chen Y., Jin M., He J., Guli A., Yan C., Ding S. (2019). Comprehensive analysis of lysine acetylome reveals a site-specific pattern in rapamycin-induced autophagy. J. Proteome Res..

[28] Xu D., Sun L., Liu S., Zhang L., Yang H. (2016). Understanding the heat shock response in the sea cucumber Apostichopus japonicus, using iTRAQ-Based Proteomics. Int. J. Mol. Sci..

[29] Zhang X., Sun L., Yuan J., Sun Y., Gao Y., Zhang L., Li S., Dai H., Hamel J.-F., Liu C. (2017). The sea cucumber genome provides insights into morphological evolution and visceral regeneration. PLoS Biol..

[30] Goodman R.H., Smolik S. (2000). CBP/p300 in cell growth, transformation, and development. Genes Dev..

[31] Grossman S.R. (2001). p300/CBP/p53 interaction and regulation of the p53 response. Eur. J. Biochem..

[32] Karamouzis M.V., Konstantinopoulos P.A., Papavassiliou A.G. (2007). Roles of CREB-binding protein (CBP)/p300 in respiratory epithelium tumorigenesis. Cell Res..

[33] Thompson P.R., Wang D., Wang L., Fulco M., Pediconi N., Zhang D., An W., Ge Q., Roeder R.G., Wong J. (2004). Regulation of the p300 HAT domain via a novel activation loop. Nat. Struct. Mol. Biol..

[34] Kim S.C., Sprung R., Chen Y., Xu Y., Ball H., Pei J., Cheng T., Kho Y., Xiao H., Xiao L. (2006). Substrate and Functional Diversity of Lysine Acetylation Revealed by a Proteomics Survey. Mol. Cell.

[35] Kovacs J.J., Murphy P.J., Gaillard S., Zhao X., Wu J.-T., Nicchitta C.V., Yoshida M., Toft D.O., Pratt W.B., Yao T.-P. (2005). HDAC6 regulates Hsp90 acetylation and chaperone-dependent activation of glucocorticoid receptor. Mol. Cell.

[36] Bali P., Pranpat M., Bradner J., Balasis M., Fiskus W., Guo F., Rocha K., Kumaraswamy S., Boyapalle S., Atadja P. (2005). Inhibition of histone deacetylase 6 acetylates and disrupts the chaperone function of heat shock protein 90 a novel basis for antileukemia activity of histone deacetylase inhibitors. J. Biol. Chem..

[37] Matthias P., Yoshida M., Khochbin S. (2008). HDAC6 a new cellular stress surveillance factor. Cell Cycle.

[38] Lee J.B., Wei J., Liu W., Cheng J., Feng J., Yan Z. (2012). Histone deacetylase 6 gates the synaptic action of acute stress in prefrontal cortex. J. Physiol..

[39] Scroggins B.T., Robzyk K., Wang D., Marcu M.G., Tsutsumi S., Beebe K., Cotter R.J., Felts S., Toft D., Karnitz L. (2007). An Acetylation Site in the Middle Domain of Hsp90 Regulates Chaperone Function. Mol. Cell.

[40] Fanghänel J., Fischer G. (2004). Insights into the catalytic mechanism of peptidyl prolyl cis/trans isomerases. Front. Biosci..

[41] Sykes K., Gething M.J., Sambrook J. (1993). Proline isomerases function during heat shock. Proc. Natl. Acad. Sci. USA.

[42] Baird T.D., Wek R.C. (2012). Eukaryotic Initiation Factor 2 Phosphorylation and Translational Control in Metabolism. Adv. Nutr..

[43] Soufi B., Soares N.C., Ravikumar V., Macek B. (2012). Proteomics reveals evidence of cross-talk between protein modifications in bacteria: Focus on acetylation and phosphorylation. Curr. Opin. Microbiol..

[44] Kamita M., Kimura Y., Ino Y., Kamp R.M., Polevoda B., Sherman F., Hirano H. (2011). Nα-Acetylation of yeast ribosomal proteins and its effect on protein synthesis. J. Proteom..

[45] Ruvinsky I., Sharon N., Lerer T., Cohen H., Stolovich-Rain M., Nir T., Dor Y., Zisman P., Meyuhas O. (2005). Ribosomal protein S6 phosphorylation is a determinant of cell size and glucose homeostasis. Genes Dev..

[46] Yang Y., Cimen H., Han M.-J., Shi T., Deng J.-H., Koc H., Palacios O.M., Montier L., Bai Y., Tong Q. (2010). NAD+-dependent deacetylase SIRT3 regulates mitochondrial protein synthesis by deacetylation of the ribosomal protein MRPL10. J. Biol. Chem..

[47] Xu D., Zhou S., Yang H. (2017). Carbohydrate and amino acids metabolic response to heat stress in the intestine of the sea cucumber *Apostichopus japonicus*. Aquac. Res..

[48] Liao G., Xie L., Li X., Cheng Z., Xie J. (2014). Unexpected extensive lysine acetylation in the trump-card antibiotic producer *Streptomyces roseosporus* revealed by proteome-wide profiling. J. Proteom..

[49] Sun L., Lin C., Li X., Xing L., Huo D., Sun J., Zhang L., Yang H. (2018). Comparative phospho-and acetyl proteomics analysis of posttranslational modifications regulating intestine regeneration in sea cucumbers. Front. Physiol..

[50] Xu Y.-X., Chen W., Ma C.-L., Shen S.-Y., Zhou Y.-Y., Zhou L.-Q., Chen L. (2017). Proteome and acetyl-proteome profiling of *Camellia sinensis* cv. ‘Anjin Baicha’ during periodic albinism reveals alterations in photosynthetic and secondary metabolite biosynthetic pathways. Front. Plant Sci..

[51] Fu L., Xu Y., Hou Y., Qi X., Zhou L., Liu H., Luan Y., Jing L., Miao Y., Zhao S. (2017). Proteomic analysis indicates that mitochondrial energy metabolism in skeletal muscle tissue is negatively correlated with feed efficiency in pigs. Sci. Rep..

[52] Apweiler R., Attwood T.K., Bairoch A., Bateman A., Birney E., Biswas M., Bucher P., Cerutti L., Corpet F., Croning M.D.R. (2000). InterPro—An integrated documentation resource for protein families, domains and functional sites. Bioinformatics.

