# Supplementary material for: Lysine Acetylation is an Important Post-Translational Modification that Modulates Heat Shock Response in the Sea Cucumber Apostichopus japonicus
Source: Int J Mol Sci. 2019 Sep 9;20(18):4423. doi: 10.3390/ijms20184423 (PMC6770049; doi:10.3390/ijms20184423)
Supplement: Supplementary file 1 [file ijms-20-04423-s001.zip › Figure S1 Predicted domains and structures of CBP.pdf]

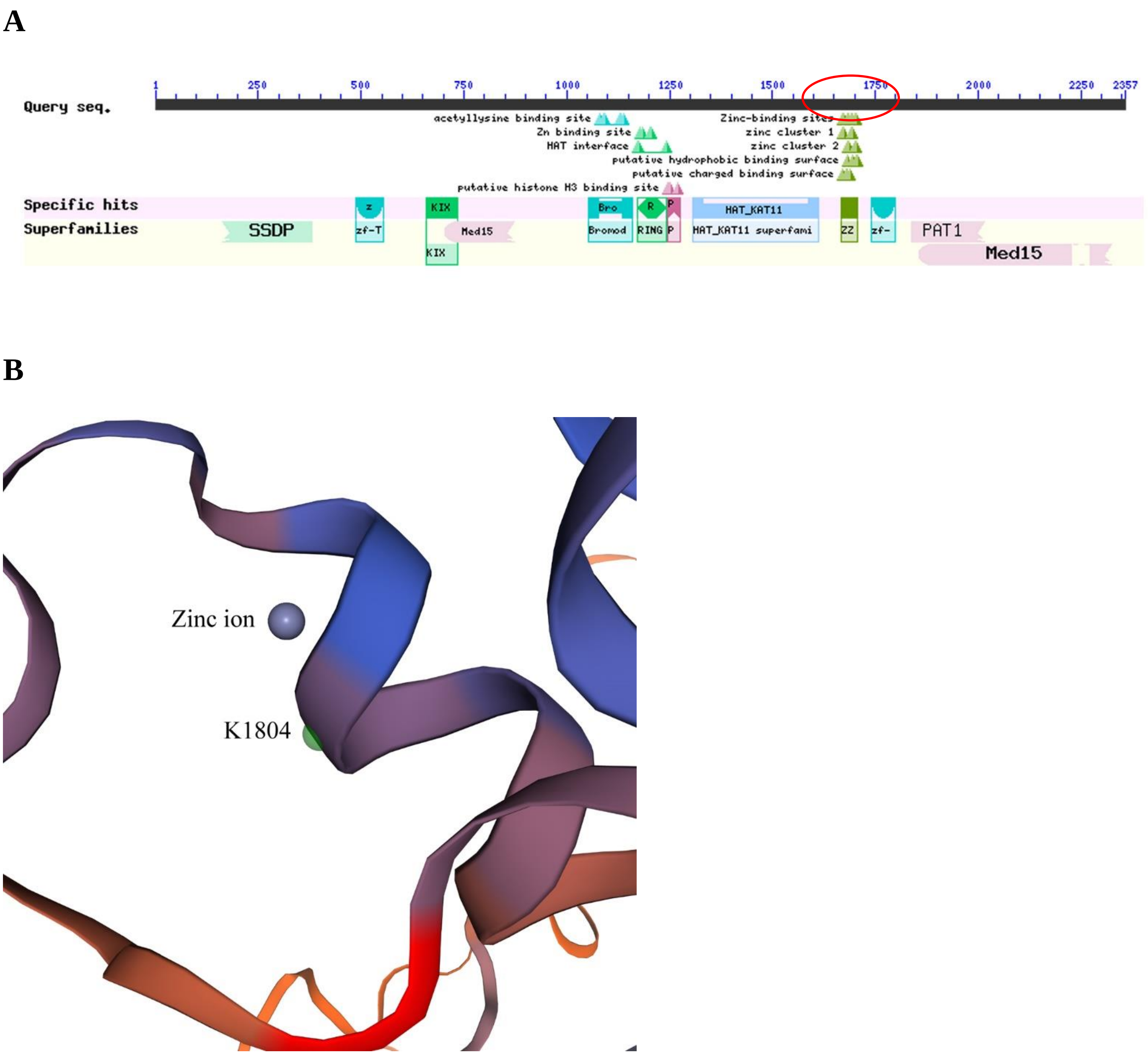

Figure S1. Predicted domains and structures of CBP. (A) The functional domains analysis of CBP in *A. japonicus* at the NCBI. The differentially acetylated lysine sites were marked by the red circle (K1583, K1590, K1766 and K1804), which were close to the predicted zinc ion binding sites. (B) Three dimensional domain structures of partial CBP predicted by the SWISS-MODEL server. K1804 was among the zinc ion binding sites.
