# Supplementary material for: Lysine Acetylation is an Important Post-Translational Modification that Modulates Heat Shock Response in the Sea Cucumber Apostichopus japonicus
Source: Int J Mol Sci. 2019 Sep 9;20(18):4423. doi: 10.3390/ijms20184423 (PMC6770049; doi:10.3390/ijms20184423)
Supplement: Supplementary file 1 [file ijms-20-04423-s001.zip › Table S2.docx]

**Table S2** **Overlapping proteins with differential quantitation of protein and acetylation sites in the HS48h vs C comparison**

| **Accession No** | **Protein name** | **Protein (log_2_ value)** | **Acetylation site (log_2_ value)** |
| --- | --- | --- | --- |
| AJAP26676 | heat shock protein 90 (HSP90) | 2.61↑ | K440, 0.96↑ |
| AJAP03428 | ELL-associated factor 2-like (EAF2) | 0.42↑ | K259, 1.13↑ |
| AJAP00341 | extracellular signal-regulated kinase 2 (ERK2) | 0.70↑ | K220, 1.24↑ |
| AJAP22233 | gamma-butyrobetaine dioxygenase (BBOX) | -0.73↓ | K479, -2.72↓ |
| AJAP10927 | phosphodiesterase family member 7-like (ENPP7) | -0.27↓ | K840, -1.01↓ |
